# Newly identified thin membranous tissue in the deep infratemporal region

**DOI:** 10.1007/s12565-012-0135-0

**Published:** 2012-04-12

**Authors:** Kojiro Takezawa, Ikuo Kageyama

**Affiliations:** Department of Anatomy, School of Life Dentistry at Niigata, The Nippon Dental University, 1-8 Hamaura-cho, Chuo-ku, Niigata, 951-8580 Japan

**Keywords:** Infratemporal fossa, Medial pterygoid, Nerve to medial pterygoid, Otic ganglion, Superior constrictor

## Abstract

Recently, the importance of deglutition has attracted attention due to its role in the prevention of aspiration pneumonia. We therefore observed the anatomy of the pharynx of 57 hemi-sections of adult Japanese cadavers (male 32 sides, female 25 sides). A previously unidentified tissue was observed in the infratemporal fossa. This unidentified tissue was a thin membranous tissue that existed between the medial pterygoid and the superior constrictor in all of the cadavers examined. The previously unknown membranous tissue consisted of collagenous and muscular fibers and was innervated mainly by a branch of the mandibular nerve.

## Introduction

Deglutition is a complicated sequence of many consecutive movements that occur in the oral cavity, pharynx, and oesophagus. Deglutition is divided into preparatory, oral, pharyngeal, and oesophageal phases. The border between the oral and pharyngeal phases is functionally and morphologically of interest as it is a border between voluntary and involuntary areas. We therefore studied in detail the deep infratemporal fossa near the adjacent parapharyngeal region. The previously unidentified tissue was discovered between the medial pterygoid and the superior constrictor. The origin, insertion and innervations of the tissue were subsequently thoroughly examined. The purpose of this study was to report on the previously unknown and newly described membranous tissue in the deep infratemporal region, and add these new anatomical findings to the study of the pharynx region.

## Materials and methods

The regions of the medial pterygoid, mandibular nerve, chorda tympani, superior constrictor, levator veli palatini, and tensor veli palatini were studied in 57 hemi-sections of adult Japanese cadavers (male 32 sides, female 25 sides) in our department at the Nippon Dental University, School of Life Dentistry at Niigata. We took samples from 26 of the same bisected cadavers. In these samples, the lateral pterygoid, medial pterygoid and the maxillary artery were dissected carefully and removed. Photographs and drawings were taken from the lateral side of the infratemporal fossa and analyzed (Canon DS126071, Tokyo, Japan). Additionally, histological structures of the tissue were observed using a polarizing microscope (Olympus BH-2 Tokyo, Japan), and photographed (Nikon E990 Tokyo, Japan).

## Results

A thin membranous structure was noted in the infratemporal fossa in all the examined cadavers. The major structures in the infratemporal fossa consist of the medial and lateral pterygoids, the mandibular nerve, otic ganglion, chorda tympani, and the maxillary artery and its many branches as well as the venous pterygoid plexus. This previously undescribed structure was located between the medial pterygoid, and the medial walls of the infratemporal fossa, which consisted of the superior constrictor and the tensor and levator veli palatini. Furthermore, this previously undescribed structure consisted of fibrous and muscular tissues, and in all cases it originated from the inferior surface of the petrous part of the temporal bone, and descended anteroinferiorly to insert into the lateral surface of the superior constrictor. In some cases, other fibers also inserted into the peripheral tissues.

**Fig. 1:**
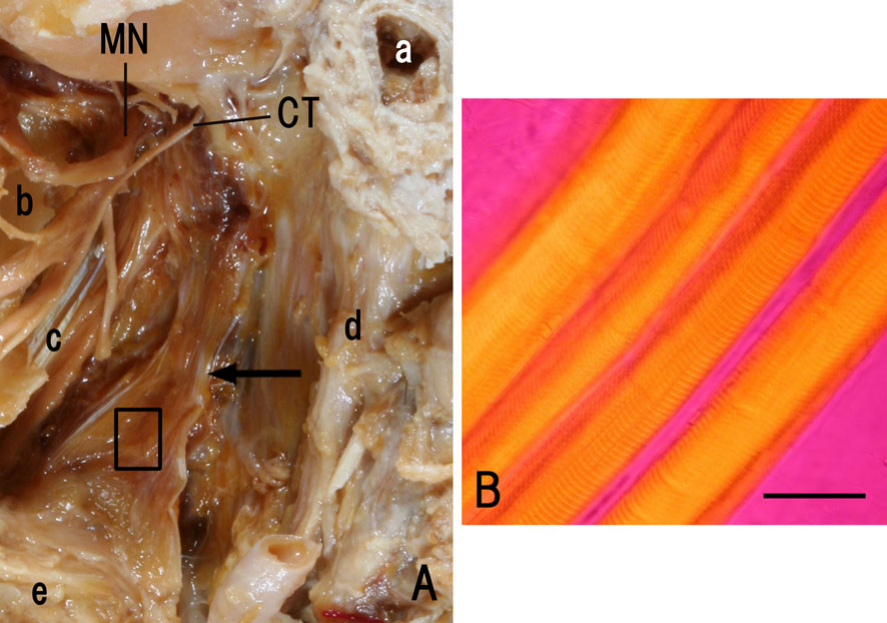
**A** Type A. Left side appearance of the infratemporal fossa of a 65-year-old Japanese man. *Arrow* Previously unidentified tissue. *CT* Chorda tympani, *MN* mandibular nerve. *a* External acoustic opening, *b* lateral plate of pterygoid process, *c* tensor veli palatini, *d* styloid process, *e* superior constrictor. **B** Polarizing microscope image, center of the box in **A**. The striated muscular fiber can be confirmed. *Bar* 50 μm

Tendinous fibers were found in the origin and insertion of the tissue (Fig. 1A), and muscular fibers (Fig. 1B) were observed by polarizing microscopy in the center of the tissue. Additionally, the tissue was classified into two types based on the pattern of insertion, into the superior constrictor or into the superior constrictor and additional tissues; namely Type A: insertion into the superior constrictor, Type B: insertion into the superior constrictor, the pterygospinous ligament, the sphenoidal bone, the buccinator, medial pterygoid, styloglossus, and mylohyoid.

**Fig. 2:**
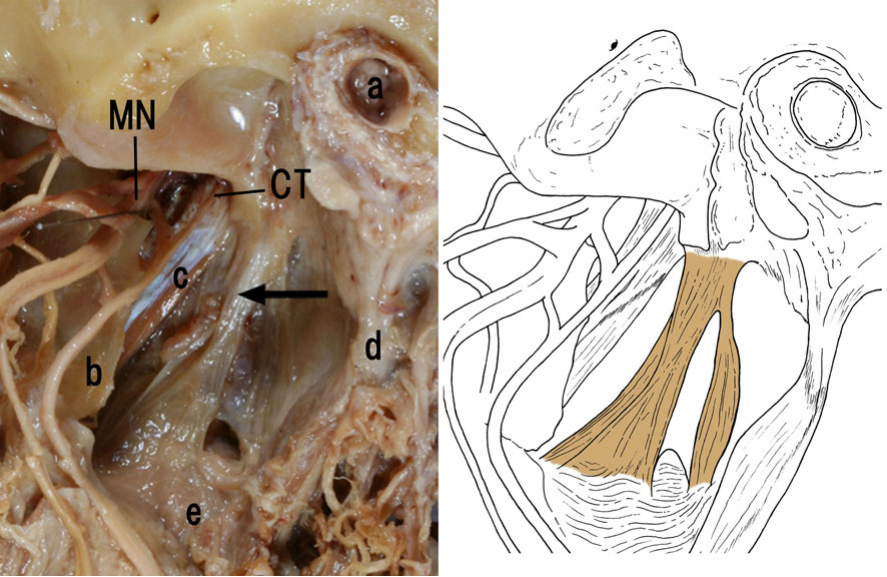
Type A. Left side appearance of the infratemporal fossa of a 75-year-old Japanese woman. *Arrow* Previously unidentified tissue. *CT* Chorda tympani, *MN* mandibular nerve. *a* External acoustic opening, *b* lateral plate of pterygoid process, *c* tensor veli palatini, *d* styloid process, *e* superior constrictor

Type A (Fig. 2) was observed in 28 cases (49 %) out of a total of 57 cadavers. The membranous tissues originated from the petrous part of the temporal bone, and inserted into the lateral surface of the superior constrictor. Type A was innervated from a branch of the nerve of the medial pterygoid in 8 out of 28 cases (29 %). One Type A case was innervated from the otic ganglion, in another Type A case was innervated from the mandibular nerve, and in two Type A cases were innervated from the branch of the submandibular ganglion. Type A innervations could not be identified in the remaining 16 (57 %) out of 28 cases.

**Fig. 3:**
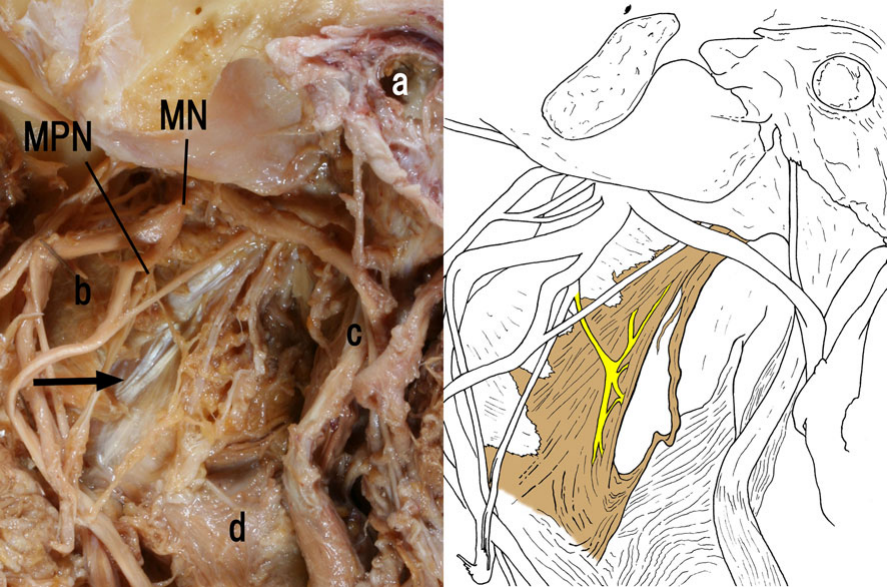
Type B. Left side appearance of the infratemporal fossa of a 85-year-old Japanese woman. *Arrow* Previously undescribed tissue. *MN* Mandibular nerve, *MPN* nerve to medial pterygoid, *a* External acoustic opening, *b* medial pterygoid, *c* styloid process, *d* superior constrictor

Type B pattern (Fig. 3) was observed in the other 29 cases (51 %) out of 57 cadavers examined. The membranous tissues originated from the petrous part of the temporal bone, as in Type A. The muscular fibers inserted mainly into the lateral surface of the superior constrictor, and in some cases inserted into the pterygospinous ligament, the pterygoid fossa, the pterygoid hamulus, the buccinator, medial pterygoid, styloglossus, and mylohyoid. Type B was observed as being innervated from a branch of the medial pterygoid in 11 out of 29 cases (38 %). In five cases out of 29, the Type B pattern was innervated from the otic ganglion, and in one Type B case was innervated from a branch of the submandibular ganglion. In the 12 (41 %) out of 29 remaining Type B cases could not be identified with their innervations.

In total, 29 (51 %) out of 57 cases of Type A and B patterns were innervated from a branch of the mandibular nerve. In 28 cases (49 %) the innervations could not be observed.

## Discussion

Our report has demonstrated that there is a previously unknown and undescribed membranous tissue that originates from the inferior surface of the petrous part of the temporal bone and inserts into the lateral surface of the superior constrictor. This tissue has not previously been reported in standard anatomy books (Bardeleben 1912; Bergman et al. 1988; Eisler 1912; Kopsch 1951; Lang 1995; Le Double 1897; Loth 1931; Quain 1914; Rouvière 1962; Toldt 1923a, b).

The adjacent tissues, namely the pharyngobasilar fascia, the tensor veli palatini, and levator veli palatini, are locate medially to the newly identified structure, whereas the medial pterygoid was located laterally. The pharyngobasilar fascia was covered by the overlapping superior constrictor, and the tensor and levator veli palatini inserted into the soft palate with the medial pterygoid inserting into the pterygoid tuberosity. The membranous tissue we report is novel, because of its insertion.

According to Albinus (Le Double 1897), the muscle originating from the petrous region and inserting into the pharyngeal muscle is referred to as the petro-pharyngeal muscle. Sandifort (Le Double 1897) reported the salpingo-pharyngen originating from the inferior part of the cartilaginous tube, and inserting into the superior constrictor. Riolan (Le Double 1897) and Macalister (Le Double 1897) reported that the spheno-pharyngen originates from the sphenoid bone, and inserts into the inferior constrictor. These muscles are the longitudinal supernumerary pharyngeal muscles. In subsequent reports, the petro-pharyngeal muscle was studied by Shimada et al. (1991). The membranous tissue observed is a different tissue, because it originates from the petrous part of the temporal bone that inserts into the lateral surface of the superior constrictor. In addition, the innervation of the present tissue differs from muscle descriptions in previous reports. Namely, the petro-pharyngeal muscles are innervated by the pharyngeal plexus of the glossopharyngeal nerve (Shimada et al. 1991); however, the newly described tissue we are reporting is innervated mainly by the mandibular nerve.

There is the possibility of this membranous tissue being divided from the tensor veli palatini or medial pterygoid. However, the insertion of this membranous tissue, the tensor veli palatini, and medial pterygoid are different in that the membranous tissue is inserted into the lateral surface of the superior constrictor, tensor veli palatini, and the medial pterygoid inserts into the soft palate and pterygoid tuberosity, respectively. Therefore, we suggest that the membranous tissue differs from these other two muscles.

If the function of this membranous tissue is considered from its origin and insertion, it is thought that it extends from the lateral wall of the pharynx superolaterally, and is involved in swallowing.

Regarding formation of the membranous tissue, the striated muscular fibers may derive from the muscle separating from mesenchymal cells of the first branchial arch. The membranous tissue described in this study was innervated mainly by a branch of the mandibular nerve. This suggests that the tissue originates from the first branchial arch. However, previous reports state that the muscles are innervated by the glossopharyngeal nerve. The glossopharyngeal nerve forms the pharyngeal plexus with the vagus nerve, showing that these muscles have a third and fourth branchial arches origin. Therefore, the tissues described in the present and previous studies are embryologically completely different. The embryological relationship between the membranous tissue and the anterior ligament of malleus or the sphenomandibular ligament was not clarified. In cases where innervation could not be identified, a more detailed study on the present tissue observed may be required for a greater understanding of the morphology, function and embryology of the pharynx. Further studies are also necessary to determine if this newly detected tissue is specific to, or if its appearance varies in, different ethnic groups.

## References

[CR1] Bardeleben KV (1912) Handbuch Der Anatomie Des Menschen, Band 2. Fischer, Jena

[CR2] Bergman RA, Thompson SA, Afifi AK, Saadeh FA (1988) Compedium of human anatomic variation. Text, atlas, and world literature. Urban and Schwarzenberg, Baltimore

[CR3] Eisler P (1912) Handbuch der anatomie des menschen, Band 2. Fischer, Jena

[CR4] Kopsch F (1951). Lehrbuch Und Atlas Der Anatomie Des Menschen.

[CR5] Lang J (1995). Skull base and related structures: atlas of clinical anatomy.

[CR6] Le Double AF (1897). Traité Des Variations Du Systéme Musculare De L’Homme.

[CR7] Loth E (1931). Anthropologie Des Parties Molles (Muscles, Intestins, Vaisseaux, Nerfs Périphériques).

[CR8] Quain J (1914) Quain’s Elements Of Anatomy, vol 2. Longmans and Green, London

[CR9] Rouvière H (1962) Anatomie Humanie Descriptive Et Topographique, Tome 1. Masson, Paris

[CR10] Shimada K, Yokoi A, Ozawa H, Kitagawa T, Tezuka M (1991). Observation of the petropharyngeal muscle in Japanese. Anat Anz.

[CR11] Toldt C (1923) Anatomischer Atlas Für Studierende Und Ärzte, Band 1. Urban and Schwarzenberg, Berlin

[CR12] Toldt C (1923) Anatomischer Atlas Für Studierende Und Ärzte, Band 2. Urban and Schwarzenberg, Berlin

